# Early Years Physical Activity and Motor Skills Intervention—A Feasibility Study to Evaluate an Existing Training Programme for Early Years Educators

**DOI:** 10.3390/children10010145

**Published:** 2023-01-11

**Authors:** Laura Basterfield, Theodora Machaira, Dan Jones, Tim Rapley, Vera Araujo-Soares, Neil Cameron, Liane B. Azevedo

**Affiliations:** 1 Human Nutrition and Exercise Research Centre, and Population Health Sciences Institute, Newcastle University, Newcastle upon Tyne NE2 4HH, UK; 2 North Yorkshire County Council, Northallerton DL7 8DD, UK; 3 SHLS Nursing & Midwifery, Teesside University, Middlesbrough TS1 3BX, UK; 4 Department of Social Work, Education and Community Wellbeing, Northumbria University, Newcastle upon Tyne NE7 7XA, UK; 5 Center for Preventive Medicine and Digital Health, Medical Faculty Mannheim, Heidelberg University, D-68167 Mannheim, Germany; 6 SportWorks (North East) Ltd., North Shields NE29 6DE, UK; 7 School of Human and Health Sciences, University of Huddersfield, Huddersfield HD1 3DH, UK

**Keywords:** early years, nursery, movement skills, physical activity, training, educators, feasibility

## Abstract

A lack of fundamental motor skills (FMS) in the early years can lead to lower engagement with physical activity (PA), and track into adulthood. This study aimed to test the feasibility of an existing intervention for Early Years Educators (“Educators”) designed to increase knowledge, confidence and the ability to increase PA and FMS of children in a deprived area of England. Non-randomised design with wait-list control. Sixty-seven settings in Middlesbrough, North East England were invited. Recruitment target: 10 settings, 2 Educators per setting, four children per Educator. Intervention: one-day training course “Physical Literacy in the Early Years”, an age-appropriate theoretical and practical training course to support the development of physical literacy. Primary outcomes: recruitment, retention, acceptability of intervention and outcome measures. Secondary outcomes: change in Educators’ knowledge, intentions and behaviour, and change in children’s BMI z-score, PA and FMS. Eight settings were recruited; all Intervention Educators completed the training. Six settings participated at follow-up (four Intervention, two Control). The target for Educator recruitment was met (two per setting, total n = 16). Questionnaires were completed by 80% of Intervention Educators at baseline, 20% at follow-up. Control Educators completed zero questionnaires. No Educators took part in a process evaluation interview. Forty-eight children participated at baseline, 28 at follow-up. The intervention was deemed acceptable. The recruitment, retention and acceptability of measurements were insufficient to recommend proceeding. Additional qualitative work is needed to understand and surmount the challenges posed by the implementation of the trial.

## 1. Introduction

There is growing evidence to support the assertion that behavioural and psychosocial experiences in early life can affect brain development and behaviour [1], and the most effective and cost-effective way to prevent health inequalities is to intervene in early life [2].

Behaviours such as physical activity (PA) bring a plethora of health benefits at a young age, including an inverse association with body fat and a positive association with better cardio-metabolic markers, bone health, psychosocial health, motor skills and cognitive development [3]. It is also known that PA can moderately track from early to middle childhood [4], showing the importance of supporting PA during the early years. Despite the well-evidenced benefits of engaging in PA [5], studies investigating PA in early years have consistently documented low PA levels [6,7,8]. However, a systematic review revealed that PA levels could vary considerably across countries, with the proportion of children meeting the recommendations ranging from 27% to 100% [9].

Lower socioeconomic status (SES) is related to lower PA levels in adults [10] and adolescents [11]. However, in young children, the association is less clear; a study of preschool children living in Scotland showed no association between socioeconomic status and accelerometer-assessed PA [12], contrasting with a study of preschool children living in deprived areas in England which found that none of the children met the recommended 180 min of PA when measured via accelerometers [13].

An increase in PA in preschool children can be achieved by improving fundamental motor skill (FMS) competence [14] with a recent meta-analysis confirming a positive cross-sectional association between FMS with moderate to vigorous and total PA in early years [4]. Equally, another systematic review showed that FMS drives PA engagement in later childhood and adolescence [15]. Early years FMS have been linked to improved cognitive, social and emotional outcomes [16]. However, there is a general concern that young children’s motor skills are declining, with a large number of studies showing that children are failing to master FMS in early years [17,18,19,20,21]. This might affect classroom learning and behaviour [22] and has important implications for health outcomes later in life, as shown in a study which reported that FMS in young children predicted health-related physical fitness at the age of 15 to 17 years [23]. It is also known that children from low socioeconomic backgrounds have a greater delay in FMS development [24] with a study of 3–5-year-old children from the North West of England showing low overall FMS competence among both sexes [20]. Morley et al. (2015) found in a sample of 4–7-year-old children, those of higher SES significantly outperformed children of middle and low SES for fine and gross motor proficiency (as assessed by the Bruininks–Oseretsky Test of Motor Proficiency, second edition brief form (BOT-2 BF) [21]). A study of Northern Irish pupils [25] in Year 1 from advantaged schools scored significantly higher in the Movement Assessment Battery for Children-2 [26] (MABC), an assessment of motor competence, than children in the same academic year from disadvantaged schools. They also found that children from disadvantaged schools were significantly more likely to have motor impairments compared to children from advantaged schools [26]. One reason could be that children living in deprived areas have limited access to safe outdoor play areas or lack opportunities to engage in activities that promote FMS [27,28].

Preschools and nurseries offer great opportunities to promote PA and FMS [29,30]. In the UK, nurseries and preschools should follow the Department for Education’s ‘Early Years Foundation Stage’ curriculum [31], where the importance of PA promotion and motor skills is reinforced. However, there is no advice and limited training on how this should be implemented. A recent systematic review highlighted that teacher-led FMS interventions could improve FMS proficiency, increase PA levels and reduce sedentary behaviour [32]. It has also been shown that FMS during childhood would primarily be acquired when children are instructed by trained specialists rather than free play time only [33]. Similarly, a critical narrative review revealed that teacher training is a vital element for successful PA interventions in preschool children [34]. Preschool teachers (Early Years Educators, “Educators”), however, have highlighted the need for more training [34], and studies focusing on teacher training for the promotion of PA in disadvantaged areas of England are limited and show no intervention effect [35,36]. Process evaluation studies targeting lifestyle interventions delivered to preschools located in deprived areas of the UK highlighted the difficulty of engaging with Educators [37], although the provision of intervention materials (e.g., classroom materials and activity guides) can facilitate the delivery of PA sessions [38]. The issues at play here are essentially ones of implementation.

To guide this study we used the Theoretical Domains Framework (TDF), which was developed through a rigorous review method and an expert consensus process [39,40]. It aggregates under domains a set of constructs across distinct behaviour theories in order to facilitate interdisciplinary communication [41]. The TDF originally grouped these constructs in 12 theoretically different constructs [42]. Following further refinement, the framework now entails 14 psychological domains of behaviour determinants. These domains are: “knowledge”, “skills”, “social/professional role and identity”, “beliefs about capabilities”, “optimism”, “reinforcement”, “beliefs about consequences”, “intentions”, “goals”, “memory/attention” and “decision processes”, “environmental context and resources”, “social influences”, “emotions” and “behavioral regulation” [40]. Both versions have been applied in research and practice across a wide range of health care settings [43,44,45], addressing problems of implementation guidelines [45] and guiding intervention development for the implementation of guidelines (e.g., [46]).

Behavioural change techniques (BCTs) [39] are intervention tools designed to target key mechanisms of action (MoA). MoA are aligned to the TDF and can target distinct domains within TDF (e.g., to target the domain Social Influences identified as a facilitator for behaviour an intervention could use the BCT 6.2. Social Comparison).

This is a feasibility study that followed the Medical Research Council (MRC) framework to design and evaluate complex interventions [47,48]. We designed a non-randomised controlled trial to test the feasibility of an intervention that teaches skills to Early Years Educators to increase children’s fundamental movement skills and physical activity. The primary aim was to understand the recruitment, randomisation, retention, fidelity, acceptability and adoption of an intervention that provides a training programme for Early Years Educators. The secondary aim was to explore the acceptability and feasibility of the potential outcome measures. Thirdly, we performed a preliminary analysis of the data to examine the effect of the intervention on knowledge gained, intention to change behaviour, BMI, FMS and PA. The ultimate aim is to collect the necessary data to design a definitive trial targeting the improvement of FMS skills and PA in pre-school children from deprived areas.

## 2. Materials and Methods

### 2.1. Ethics Statement

Ethical approval for the study was granted by the Newcastle University Faculty of Medical Sciences Ethics Committee (01223/9764/2016). Data collection took place between March 2017 and February 2018.

Clinicaltrials.gov identifier: NCT04868084 30 April 2021, Retrospectively registered https://clinicaltrials.gov/ct2/show/NCT04868084?term=NCT04868084&draw=2&rank=1 (accessed date 9 February 2021).

### 2.2. Study Design

This was a non-randomised [49] before-and-after study design (clustered) with wait-list control (i.e., the Control group received the same intervention after the completion of the study). Due to a delay in the recruitment process and a low uptake, nursery settings (“settings”) were not randomised to Intervention or Control arms as had been originally planned. Instead, the first settings that agreed to participate in the study were placed in the Intervention arm and the next in the Control arm. However, settings were broadly equivalent for the relative deprivation of the neighbourhood in which they were situated (i.e., all at the bottom decile for the Index of Multiple Deprivation (IMD)) [50].

### 2.3. Setting Location

Data collection took place between March 2017 and February 2018. All nurseries and nursery schools in the Middlesbrough area were eligible to take part in the study. Middlesbrough is a densely populated and ethnically diverse town in the North East of England and the sixth most deprived local authority in the country according to the Index of Multiple Deprivation (IMD) [50]. More than half of the children in Middlesbrough (63%) live in the top 10% most deprived wards in the country [51]. One in four reception class children (27.1%) were categorised as living with overweight or obesity in Middlesbrough compared with one in five (21.9%) for England [51]. Children in Middlesbrough are also less likely to achieve a good level of school readiness (children’s competencies when they enter school, measured by communication and language skills, physical development, personal, social and emotional skills and academic skills such as mathematics and literacy [52]) compared to the England average (60.3% Middlesbrough vs. 69.3% England) [53].

For a better understanding of the socio-demographics of the recruited settings, two markers of deprivation were included: IMD rank and decile, and free school meals eligibility. The IMD from small areas are ranked from 1 (most deprived) to 32,482 (least deprived) [50]. The deciles are calculated by ranking these small areas in England from most deprived to least deprived and dividing them into 10 equal groups. Also, in England, children from families in receipt of a number of income-related welfare benefits are entitled to free school meals.

### 2.4. Recruitment

We aimed to recruit 10 settings to the study and include two Educators from each setting. Letters were sent to the managers of all settings in Middlesbrough, who looked after children aged 3–4 years. This was followed by a phone call to discuss the study further. Managers provided written consent for their setting to be involved. Schools’ Head Teachers or nursery managers acted as a gatekeeper and gave the information sheet to all Educators caring for this age group at their setting. A maximum of two participants per setting were randomly selected and provided written consent for their participation. All the children who were cared for by those staff members were eligible to participate, with a maximum of four recruited (selected at random should more than four parents have given consent). Parents were given written information about the study, and the opportunity to ask the researchers any questions. Parents willing to participate provided written consent for their child’s participation. Children had the study explained to them and gave their verbal assent to participate. They were told that they could stop taking part at any time.

### 2.5. Intervention

The intervention’s overarching objective was to improve the Educators’ ability (that could be increased through further knowledge and skills provision) and confidence (believe in their own capability to do so) to teach children’s fundamental movement skills and increase children’s PA levels. Educators were offered a one-day course which was based on a whole-systems approach, and focused on creating enabling environments and planning PA for all developmental stages (for all children). It offered a balance between child-initiated and adult-led PA to promote PA beyond the nursery setting (reaching the home setting).

The ‘Physical Literacy in the Early Years’ training course (“intervention” for the purpose of this manuscript) was delivered by an external provider, SportWorks [54] on 6 May 2017. The intervention was delivered by a physical activity specialist with background in both theoretical aspects of early age health interventions (university lecturer) and practical implementation within localised settings (director of sports development company specialising in community health provision within the UK). The training was delivered to Educators from the Intervention settings in a six-hour session on a weekend (non-work time) at Teesside University. The training provided Educators, teaching assistants and others working with children under five years the knowledge (BCT: instruction on how to perform a behaviour), skills (BCT: Behavioural Practice/Rehearsal) and confidence (BCT: Graded Tasks) to deliver enjoyable and engaging lessons which focus on the development of core “fundamental” motor skills. The course was developed in conjunction with leading physical education consultants, sports scientists and Office for Standards in Education and Children’s Services and Skills (Ofsted) advisors. The course covered the theory about FMS and physical literacy and offered practical demonstrations of age-appropriate ways to teach and develop FMS. Details are as follows:

The training (intervention) that was delivered to those in charge of the interaction with the children was structured into two parts. First, a three-hour theory-based workshop was conducted within a classroom setting. The session was structured based upon a tutor-led, interactive “workshop” model in order to raise awareness and understanding of “physical literacy” for Educators. The workshop incorporated the following subject areas: the definition of the terms “physical literacy”, “fundamental movement skill” and “gross motor skill” to raise Educators’ understanding and knowledge base (targeting the shaping of knowledge); an overview of key scientific literature, data and trends to establish a rationale for investing time in addressing childhood obesity, physical activity and childhood development/maturation (targeting intention/motivation/goal setting); self-reflection of Educators’ existing work and teaching methods in relation to physical education, exercise and education (targeting self-monitoring of behaviour); a review of physical and environmental factors which affect children’s engagement and sustained participation in exercise (e.g., increasing understanding of environmental contexts and resources); and a review of good practice guidelines and recommendations for the delivery of physical education and physical literacy within the early years and educational environments (N.B. content adopted from Department of Education School Curriculum for Physical Education (Key Stage 1) and Association for Physical Education (AfPE)).

Following a 60 min lunch break, the second part of the intervention was delivered in the afternoon in a sports hall environment. The two-hour session was structured within a ‘practical’ format (modelling and prompt practice were used as Behaviour Change Techniques [55]) and Educators requested to wear appropriate exercise/leisure clothing. The workshop incorporated the following subject areas:

1. Practical overview of children’s physical developmental phases at 12 months, 18 months, 24 months (2 years of age), 36 months (3 years of age) and 60 months (5 years of age) in order to identify typical changes in movement proficiency over time (provision of knowledge);
2. An introduction to practical physical literacy included delivery considerations and good practice guidelines (content based upon UK Athletics’ ‘Run, Jump, Throw’ curriculum [56] and ‘Fundamental Motor Skills: A Manual for Classroom Teachers’ (1996) [57];
3. Introduction to, and practical demonstration of, a range of physical activities suitable for early years Educators and settings (modelling with prompt practice). Main movement patterns reviewed and demonstrated included:
  - Crawling, walking, running, hopping, leaping, galloping, dodging, swimming;
  - Balance, spatial awareness, linking of movements;
  - Object control;
  - Summary of the Department for Education’s Physical Education curriculum and Ofsted’s assessment guidance regarding the practical delivery of physical education lessons.

Educators were each required to plan and deliver a 10 min practical lesson based upon aforementioned content (goal setting, action planning, prompt practice). Three children (all aged 5 years or under) were in attendance and took part in activities.

V. Review (feedback on the behaviour), reflection and action planning

The course provided knowledge and resources which could be directly applied to each setting, leading participants through the necessary principles/knowledge, skills and applications of physical literacy for successful planning of activities/classes and teaching/engagement with their pupils. Educators gave feedback on the course via a 16-item questionnaire. After the training session, Educators were encouraged to speak with the training provider to follow up on their understanding of the training and to gather, if needed, additional help for implementation of the practices learned. Control settings continued their usual practice and were offered the intervention at the end of data collection.

Staff from all settings were paid for their time: GBP 50 for attending the training course and GBP 20 for responding to the questionnaires. Nurseries received a payment of GBP 100 as thanks for participation.

### 2.6. Outcomes

The primary study aims were to assess the recruitment, randomisation, retention, fidelity and acceptability of the intervention. Therefore, the following primary outcomes were assessed:

(1) Recruitment rate, which includes consented participants to potentially eligible participants approached;
(2) Compliance and acceptability of the intervention was measured by (a) the number of participants attending ‘Physical Literacy in the Early Years’ intervention training course, and (b) direct observation of the Educator by one of the researchers in the settings for 30 min by using an adapted form of the Communication Supporting Classroom Observation Tool [58].

The secondary aim was to explore the acceptability and feasibility of potential measures that would assess changes in the targets of the intervention, and to address this we piloted some measures which could be considered in a definitive trial to assess intervention effectiveness. These included:

(1) Knowledge gained from the intervention assessed by knowledge of the PA guidelines and benefits for young children using a 16-item questionnaire (Additional File 1a);
(2) Behaviour Change Domains assessed by a 50-item questionnaire adapted from Huijg et al. (2014) [59]. The questionnaire covered the following TDF [40] domains: Knowledge; Skills; Social/Professional Role and Identity; Belief about Capabilities; Optimism; Beliefs about Consequences; Intentions; Goals—Action Planning; Goals—Priorities; Memory, Attention and Decision Processes; Environmental Context and Resources; Social Influences; Emotion; Behavioural Regulation; Social and Professional Role; Work Environment (Additional File 1b);
(3) Children’s anthropometric measures including height (to 0.1 cm) and weight (to 0.1 kg) which were measured twice without socks or shoes, in indoor clothing, using a portable stadiometer (Leicester Height Measure, Child Growth Foundation, London, United Kingdom) and calibrated scales (Seca 761, Seca Weighing and Measuring Systems, Birmingham, England). Body mass index (BMI) was then derived, and z-scores calculated relative to UK1990 data [60];
(4) Children’s PA was measured directly using a thigh-mounted accelerometer (activPAL) for 7 days. The activPAL is a uniaxial accelerometer that classifies movement and postures into sitting or lying, standing and stepping and is valid, reliable and well-tolerated by young children [61]. The activPAL was worn in the middle of the anterior aspect of the right thigh attached using a waterproof medical grade adhesive dressing (3M Tegaderm), and worn underneath normal clothes. Data were collected in 15-s epochs, and non-wear time was defined as 10 min of consecutive zero counts and removed from daily wear time. Children were asked to wear the monitors while at the setting. Analyses of PA data (recorded with activPAL) were undertaken using percent of wear time in each of the postures (i.e., sitting, standing and stepping) during school or nursery hours. The use of percentage of wear time accounts for potential differences in wear time within and between participants;
(5) Fundamental motor skills were assessed with the Movement Assessment Battery for Children-2 (MABC-2) [26]. The MABC-2 assesses motor skill proficiency in three domains: (i) manual dexterity (fine motor skills); (ii) aiming and catching (object control skills), and (iii) balance. The raw scores for each task were converted into age standardised scores, which were then summed to give a domain score. The standard score for each domain was again summed to give a total standard score [26];
(6) Compliance and acceptability of these measures was assessed by the number of participants completing them at baseline and follow-up.

### 2.7. Statistical Analysis

Descriptive data were processed for the following primary outcomes: (1) recruitment ratio (ratio of consented participants to potentially eligible participants approached) and (2) compliance with, and adoption of, the intervention (numbers attending the intervention and numbers of observed PA and FMS activities performed by the Educators).

Basic descriptive data were generated for all secondary outcomes: numbers completing the measures, knowledge gained from the intervention, behaviour change domains, BMI, FMS and PA. Although this was a feasibility study, with a relatively low sample size, inferential statistics were performed to provide preliminary information about potential effectiveness of the intervention. Dependent *t*-tests were used to explore the difference between follow-up and baseline on children’s anthropometrics, fundamental motor skills and PA data. All analyses were conducted using SPSS v.14.

## 3. Results

### 3.1. Primary Outcomes

#### 3.1.1. Participants and Recruitment

Of the 67 settings (nurseries n = 34, primary schools with attached nursery n = 33) that were invited to participate, 10 positive responses were received, of which eight entered the study for baseline measures (i.e., the target of 10 settings was not met). One non-participating setting was mistakenly invited but was outside the geographical region, and in the other non-participating setting the nursery manager agreed to participate but was unable to get any parents to consent and consequently no Educators participated either. From those eight settings, six completed follow-up measures. Table 1 presents information describing the settings recruited. Five settings were placed in the Intervention and three settings in the Control arm. Intervention and Control arms were broadly equivalent in terms of total number of children enrolled, index of multiple deprivation and free school meals. Generic data (range of IMD rank and decile and free school meal eligibility) from eligible settings that did not agree to participate are also presented. A total of 46/48 children spoke English as their first language, but we do not have information on the age/ethnicity/level of education of the Educators.

#### 3.1.2. Acceptability of the Intervention

Two Educators were recruited from each setting (100% of target), and all of the Intervention Educators completed the intervention training course. All Educators who completed the intervention training found the course interesting and gained new knowledge. All found the trainer knowledgeable and approachable, and 9/10 thought the content was delivered at the right speed. Eight out of 10 were happy to attend at the weekend, with only one participant not wanting to attend and the other did not respond. Eight out of 10 said they would have attended if they were not getting paid, two participants did not respond. Eight out of ten would recommend the course to colleagues, while one said that they would not and the other did not respond. Although the study was wait-list control, and the training course was offered to all Control settings at the end of the data collection period, none of the Control settings took up this offer after being contacted four times. Likewise, the training provider tried to engage with participants who took part in the intervention to follow-up on the understanding of the training but none of the intervention participants (n = 10) returned the phone calls. Finally, none of the Educators (n = 16) agreed to take part in a focus group or interview to explore reasons for the lack of interest and drop-out rate from either Intervention or Control Educators or settings.

#### 3.1.3. Participant Retention, and Acceptability of Potential Secondary Outcome Measures

In terms of meeting the primary outcomes of the study, 4/5 of Intervention and 2/3 of Control settings were involved in at least some of follow-up measures (Table 2). Questionnaires exploring the intentions for behaviour change were completed by 10/10 Intervention and 0/6 Control Educators at baseline, and by 2/10 Intervention and 0/6 Control Educators at follow-up. Parental consent and child assent were provided by 35 Intervention children and 13 Control children at baseline and at least one measure was recorded (anthropometric, FMS or accelerometry) (Table 2). One intervention setting dropped out before follow-up measures, and all the children from one Control setting had left the setting (to start school) before follow-up.

#### 3.1.4. Observations of Educator Practice

Results of Educator observations are presented in Supplementary Table S1. Although Educators taking part in the study were the ones who were focused on, all staff in the room were observed, whether in the study or not. This was due to more staff being present to ensure the correct ratio of staff : children were maintained, and all staff in the room were therefore interacting both with each other and with the children. All observations were performed at baseline only, due to Educators not engaging at follow-up, so we are unable to offer an assessment of whether the intervention altered Educator behaviour. Educators were generally observed to encourage activity and movement, but those requiring more specialised guidance and co-ordination, such as hopping, jumping and skipping, were observed less often. However, a longer observation time may have changed this.

### 3.2. Secondary Outcomes

#### 3.2.1. Educators’ Change in Knowledge

Seven Intervention Educators completed the questionnaire at baseline, two at follow-up. At baseline, 4/7 correctly answered the question on what the PA guidelines for under 5s are. At follow-up, the two responders who had previously offered incorrect answers, gave the correct answer. At baseline, 3/7 had heard of “fundamental movement skills”. At follow-up this was 2/2 (both had responded “no” at baseline). Examples they gave of what they thought it meant were generally correct. At baseline, 1/7 had heard of “physical literacy”. At follow-up this was 2/2 (both had responded “no” at baseline), however no answers really captured what this is (although this is not surprising as it is a difficult concept to articulate and the definition is quite long). At baseline, 5/7 respondents gave examples of benefits to young children of being active, although some went into adulthood. At follow-up, 2/2 gave appropriate examples (one had not answered this question at baseline).

#### 3.2.2. Educators Change in Behaviours

The questionnaire on Educator intentions and beliefs at baseline was completed by 10/10 Intervention Educators but 0/6 of the Control Educators; results are in Supplementary Table S2. Educators rated themselves as above average for most of the domains, with Environmental context and resources (i.e., the nursery setting), being a notably low exception. None of the Educators from either Intervention or Control completed this questionnaire at follow-up.

#### 3.2.3. Anthropometric, Physical Activity and Fundamental Motor Skills

The children’s results are not discussed in detail as the study was not powered to make those comparisons. However, data presented in Supplementary Table S3 show that age, gender, anthropometric, PA and fundamental motor skills were similar between Intervention and Control groups at baseline and follow-up. Changes from baseline to follow-up from the Intervention group are presented in Supplementary Table S4. The results show that there was a significant increase in weight and a significant decrease in percentage of time stepping.

## 4. Discussion

This study aimed to test the feasibility of using an existing training course for Early Years Educators to increase their knowledge of, and confidence in, delivering PA to young children. We focused on nursery settings in a deprived area of the UK in order to target health inequalities since children living in these areas show a delay in FMS development [20,24].

Our feasibility study produced mixed results in terms of recruitment, retention and acceptability. Recruitment was challenging, and two nurseries (one from the intervention group and one from the control group) dropped out at follow-up, showing difficulty retaining these settings. However, Educators and children accepted the intervention well, and all Educators believed they gained knowledge from the course. Some challenges were also noted in the acceptability of measurements, particularly for the control group.

### 4.1. Recruitment and Retention

The initial recruitment took longer than expected, leading to delays in the start date for baseline measures and the delivery of the Intervention training course, which led to delayed follow-up. A good proportion of parents agreed to give consent for their child (except for one setting). However, because of the delays some of the older children had left the nursery setting to start school, which affected retention.

The stressful environment in which Early Years Educators are operating might have contributed to the unsuccessful recruitment and retention of Educators and managers in our study. Additionally, the challenge of working in deprived communities is not new, as summarised by White et al. [62], together with suggestions for increasing recruitment/retention [62]. This is increasingly of concern as the inequality gap widens and there should be an attempt to focus on representation from participants in those communities [63].

Similar to our findings, a study that targeted lifestyle behaviours in preschool children and their families performed in North East England reported a similar recruitment rate (10% vs. 12%) and struggled to engage Educators [37]. The authors suggested a possible lack of information transfer from the consenting nursery manager to the Educators since this ‘buy-in’ from the staff who are actually working with the children is crucial to the success of the study. On the other hand, a preschool PA intervention facilitated by a member of school staff in a deprived and multi-ethnic population in the North West of England was feasible concerning the recruitment and retention of children [64], showing the importance of a key contact or champion for access and endorsement [65]. Similarly, issues on recruiting and retaining participants were overcome in a recent feasibility study performed in South West England [66], which tested an amended version of an existing American intervention (Nutrition and Physical Activity Self-Assessment for Childcare (NAP SACC)) [67]. Delivered and supported by Health Visitors, the multi-component study successfully recruited from high/medium/low areas of deprivation. The support of Health Visitors might have contributed to the successful recruitment and retention of nurseries in that study, a theory supported by a previous nursery intervention targeting oral health [68]. This could indicate a strategy for consideration in future studies. Other strategies to increase the retention of participants throughout the study might include offering the control group another type of health intervention (e.g., immunisation, dental health) and additional intervention contact time through newsletters. Such strategies appeared successful in retaining Latino children and their parents in an obesity prevention intervention [69].

### 4.2. Acceptability of the Intervention and Measurements

Once recruited, Educators in the Intervention group reported that they enjoyed and gained knowledge from the training. However, they have not further engaged with the training provider after several attempts to contact them by phone. Likewise, children also appeared to have enjoyed being involved in the intervention.

The acceptability of the measurements to meet the secondary aims was mixed. Baseline measures and some follow-up measures were completed in the Intervention group. However, Control Educators were not engaged and did not complete the questionnaires at baseline or follow-up. Unfortunately, no Educators from either group agreed to participate in the interviews or focus group discussions, so we have no direct evidence for their reasons.

Many reasons could have contributed to the lack of success in engaging this group following the intervention and measurements. The early years’ workforce has been facing difficulties for many years, which were compounded by the COVID-19 pandemic. As reported recently by the UK Social Mobility Commission [70], recruitment and retention of staff in early years settings is a challenge, with one in six staff in the UK opting to leave within one year [71]. The poor working conditions, including low wages, long hours, lack of progression and low recognition, are some of the reasons why many highly qualified professionals leave the sector for better career opportunities. In addition, a lack of training and continuous professional development affects their qualifications and progression and might have a long-term impact on young children’s education and school attainment [72]. The situation is more unfavourable in deprived areas, with recent evidence that nurseries in deprived areas in the UK face closure over the funding gap [73]. Consequently, this could widen the educational gap between children from rich and poor backgrounds as there is evidence that high-quality childcare is associated with cognitive, language and social development benefits, particularly for children from disadvantaged backgrounds [72].

Options to support long-term sustainability and reduce costs could be the delivery of the intervention by teachers or childcare staff, as reported by Barber et al. 2016 [64]. However, this might not be effective. A systematic review that explored the effect of centre-based childcare PA interventions revealed that interventions delivered by teachers or staff did not promote significant changes in PA. However, there was a positive effect when experts (such as active play professionals or sports coaches) or external staff (such as Health Visitors) delivered the intervention [74]. The same systematic review showed that pragmatic interventions (i.e., delivered under ‘real-world’ conditions) did not have a significant effect, while non-pragmatic interventions were effective. The authors suggested that this might be related to the fact that staff had difficulty implementing the intervention with fidelity which could then impact effectiveness [74].

As noted earlier, parents were receptive to providing their consent for their children to participate in this study. This might be related to the fact that this intervention required minimal engagement of parents (i.e., parental consent for measurements on children). According to the socio-ecological model, parents and children would be the most influential and proximal system to affect children’s PA behaviour, although evidence of using this approach for PA in early years is weak [16,75]. It is unclear how a more intense parental engagement in the intervention would affect the feasibility of the study, as parents living in deprived areas in Wales reported a range of community, social, economic and personal barriers to promoting PA in young children [76]. There might also be an additional challenge to working with children from deprived areas, as a systematic review that explored PA interventions in socio-economically disadvantaged communities showed that although PA interventions are effective for adults, they were not for children [77].

The focus on an FMS intervention for this particular population is relevant as it is known that socioeconomic status is associated with motor competence (children from higher SES backgrounds had better motor competence) [78]. Likewise, there is a decline in motor competence in children starting school [79], with 32.2% of children starting school with or at risk of having a movement difficulty, a figure that has approximately doubled in 10 years (2007–2017). Nearly all teachers from a UK survey (96%) believed that children were starting school less physically prepared than in previous years [79]. In the US, a community-based intervention delivered by teachers four days a week with booster sessions supported by research students found that the effects of increasing FMS in preschool children from deprived communities could be long-lasting [80]. However, children may also improve FMS using a ‘light touch’ approach of weekly exercise and games and environment restructure [81].

Anecdotally, the Educators involved in this study were themselves inactive, which might have affected the ‘buy-in’ for a physical activity intervention. Perhaps the intervention needs to include a focus on the lifestyle behaviours of the Educators themselves, in particular their physical activity. Beliefs about capability, of which self-efficacy and confidence in one’s own skills are an integral part, are also critically important for Educators in early years settings. A previous qualitative study with Educators, mentors and managers highlighted that developing confidence through taking part in an educational programme is vital to contribute to the sense of ‘being’ a professional [82]. Strategies to increase the physical activity of Educators and build confidence might help overcome the perceived barriers, including lack of time and motivation [83].

### 4.3. Strengths and Limitations

There were positives to take from the study: the intervention (training course) was reported as enjoyable and informative by the Educators, parents at each setting consented to their child’s participation and children were willing to be involved. However, there were several unsurmountable challenges for a small feasibility study, including but not limited to children leaving settings, children being absent on testing days, staff turnover, change in management, non-return of telephone calls by both settings and Educators and lack of Educator engagement. The timing of the study is crucial, as children leave nursery settings to move on to school. Delays in recruitment or being able to take follow-up measures will impact study retention and lead to missing data.

### 4.4. Summary and Recommendations

In summary, the recruitment and acceptability of outcome measures and intervention were relatively successful for parents and children, and the training element of the intervention was well-received by the Educators. However, the same was not observed for the recruitment of nursery practices and the acceptability of the secondary outcome measures for Educators. Although the aim of this study was not to explore the effect of the intervention on health-related outcomes, it is likely that the poor recruitment, retention and acceptability of the measures would negatively impact the outcomes and benefits of the intervention.

Several strategies could be employed to increase the participation of settings and Educators:

- Use of a co-production approach with the Educators and managers might be effective in designing the intervention and measurements. It could provide a sense of ownership, increase engagement and might help to overcome barriers [84];
- Further involvement and incentives of local government (e.g., UK Local Authorities) could support a more coordinated approach across local nurseries, in addition to shared values with decision makers, which is important for intervention success and sustained impact [85];
- Include gatekeepers to facilitate recruitment [86], not only to grant researchers access but to legitimise the researchers’ role. A recent study used lay advisors affiliated with the university to undertake community recruitment [87], and stressed the effort that went into forging relationships with gatekeepers;
- Explore other delivery modes through Health Visitors, sports coaches and play professionals, for example, as this has been successful previously [66]. However, the long-term effectiveness of this approach is questionable, especially with the many competing demands of these professionals;
- Use of financial compensation, which may aid retention [87]. There is evidence that financial incentives might increase participation in low-income families of young children [88]. However, financial incentives did not have a noticeable impact on the current study, although recruitment may have been lower without one. Other studies have reported that financial incentives might have a negative connotation in some cultures [89], while others raised ethical concerns about offering incentives for research to disadvantaged people [90].

## 5. Conclusions

Engaging with preschool settings and Educators has many challenges, especially in a deprived area, and may explain the failure of this and previous interventions. Future preschool interventions in this region should seek the involvement of the settings and Educators in the design and development of the intervention as much as is practicable and consider other strategies, such as the involvement of policy decision makers and other health professionals.

## Figures and Tables

**Table 1:** Demographics of Intervention and Control settings (index of multiple deprivation (IMD), free school meals and children recruited).

Schools	IMD Rank (Decile) ^1^	Free School Meals Eligibility (%)	Children Recruited (n)
Intervention:			
1	68 (1)	47	7
2	8905 (3)	39	8
3	59 (1)	59	7
4	376 (1)	70	5
5	24,695 (8)	13	8
Control:			
1	9247 (3)	12	6
2	799 (1)	NA ^2^	4
3	613 (1)	NA ^2^	4
Eligible but NOT recruited	26 to 31,525 (1 to 10) ^3^	1 to 57	-

^1^ 1 = most deprived. ^2^ NA = not available. ^3^ unit (range).

**Table 2:** Numbers of settings/Educators/children involved in the study.

	Intervention (n)		Control (n)	
	Baseline	Follow-Up	Baseline	Follow-Up
Settings				
Participation	5	4	3	2
Observations	5	0	3	0
Staff				
Participation	10 ^1^	2	6	0
Questionnaires	10	2	0	0
Interviews	0	0	0	0
Children				
Total consent given	35 (46% female)	-	13 (38% female)	-
Completed:				
Anthropometry	30 (40% female)	23 (35% female)	13 (38% female)	5 (100% female)
FMS	30 (40% female)	23 (35% female)	13 (38% female)	5 (100% female)
Accelerometry (activPAL)	21 (42 % female)	15 (36% female)	6 (40% female)	0

^1^ All completed training.

## Data Availability

Data are being prepared for archiving, and until then are available from the lead researcher upon request.

## Supplementary Materials

The following supporting information can be downloaded at: https://www.mdpi.com/article/10.3390/children10010145/s1. Additional file 1a: knowledge questionnaire; Additional file 1b: implementation questionnaire; Supplementary Table S1: observations of educator behaviour at baseline; Supplementary Table S2: results of the questionnaire measuring Educator behavioural predictors at baseline (completed by Intervention Educators only, n = 8); Supplementary Table S3: participants’ characteristics (children) at baseline; Supplementary Table S4: intervention group comparison follow-up at baseline.
