# Supplementary material for: Early Years Physical Activity and Motor Skills Intervention—A Feasibility Study to Evaluate an Existing Training Programme for Early Years Educators
Source: Children (Basel). 2023 Jan 11;10(1):145. doi: 10.3390/children10010145 (PMC9856565; doi:10.3390/children10010145)
Supplement: Supplementary file 1 [file children-10-00145-s001.zip › children-2097562-Additional_File_1a_knowledge_Questionnaire.pdf]

## Open questions practitioner’s knowledge questionnaire

Thank you for taking part in our study. We appreciate you completing the following questionnaire. Your answers will be kept confidential. If you have any queries, please speak to one of the researchers.

The UK government issues recommendations on the amount of physical activity the population should take part in. What are the current UK recommendations for physical activity for under 5s who can walk?

……………………………………………………………………………………………………………………………………………………

……………………………………………………………………………………………………………………………………………………

Have you heard of the term ‘fundamental movement skills’?

☐ Yes

☐ No

What do you understand by the term ‘fundamental movement skills’?

……………………………………………………………………………………………………………………………………………………

……………………………………………………………………………………………………………………………………………………

Have you heard of the term ‘physical literacy’?

☐ Yes

☐ No

What do you understand by the term ‘physical literacy’?

……………………………………………………………………………………………………………………………………………………

……………………………………………………………………………………………………………………………………………………

Do you know any benefits to young children being physically active? Can you list them here please?

………………………………………………………………………………………………………………………………………………………

………………………………………………………………………………………………………………………………………………………

## Question to be added for the intervention group after 6 months of intervention

Do you think the training has increased your confidence and ability to provide developmentally appropriate physically active play for the children you care for?

☐ Yes

☐ No

☐ Not sure

Could you please provide examples on aspects when and where your confidence has improved?

………………………………………………………………………………………………………………………………………………………

………………………………………………………………………………………………………………………………………………………
