# Supplementary material for: Early Years Physical Activity and Motor Skills Intervention—A Feasibility Study to Evaluate an Existing Training Programme for Early Years Educators
Source: Children (Basel). 2023 Jan 11;10(1):145. doi: 10.3390/children10010145 (PMC9856565; doi:10.3390/children10010145)
Supplement: Supplementary file 1 [file children-10-00145-s001.zip › children-2097562-Additional_File1b_Implementation_questionnaire.pdf]

Implementation questionnaire

Please read each question with care and answer each question with an X on a box (1-7) . Please answer what first comes to your mind. There is no right or wrong answer but only what is important for you. Thank you for your collaboration.

1. I am aware of the contents and objectives of physical activity guidelines for early years.

1	2	3	4	5	6	7
Strongly Disagree						Strongly Agree

2. I know the contents and objectives of physical activity guidelines for early years.

1	2	3	4	5	6	7
Strongly Disagree						Strongly Agree

3. I am familiar with the contents and objectives of physical activity guidelines for early years.

1	2	3	4	5	6	7
Strongly Disagree						Strongly Agree

4. I am aware of how to implement physical activity in my nursery/school with young children when at work.

1	2	3	4	5	6	7
Strongly Disagree						Strongly Agree

5. I have undertaken training on how to implement physical activity in my nursery/school when at work with young children.

1	2	3	4	5	6	7
Strongly Disagree						Strongly Agree

6. I have the skills to implement physical activity in my nursery/school when at work with young children.

1	2	3	4	5	6	7
Strongly Disagree						Strongly Agree

7. I have practiced implementing physical activity in my nursery/school when at work with young children.

1	2	3	4	5	6	7
Strongly Disagree						Strongly Agree

8. Implementing physical activity in nursery/school when at work with young children is part of my work as an educator.

1	2	3	4	5	6	7	
Strongly Disagree						Strongly Agree	

9. As an early years educator it is my job to implement physical activity in my nursery/school when at work with young children.

1	2	3	4	5	6	7
Strongly Disagree						Strongly Agree

10. It is my responsibility as an early years educator to implement physical activity in my nursery/school when at work with young children.

1	2	3	4	5	6	7
Strongly Disagree						Strongly Agree

11. Implementing physical activity in early year settings when at work with young children is consistent with my early years educator role.

1	2	3	4	5	6	7
Strongly Disagree						Strongly Agree

12. I am confident that I can implement physical activity in my nursery/school when at work with young children even when young children are not motivated.

1	2	3	4	5	6	7
Strongly Disagree						Strongly Agree

13. I am confident that I can implement physical activity in my nursery/school when at work with young children even when there is little time.

1<br>Strongly<br>Disagree	2	3	4	5	6	7<br>Strongly<br>Agree

14. I am confident that if I wanted I could implement physical activity in my nursery/school when at work with young children

1<br>Strongly<br>Disagree	2	3	4	5	6	7<br>Strongly<br>Agree

15. With regards to implement physical activity in my nursery/school when at work with young children in uncertain times, I usually expect the best.

1<br>Strongly<br>Disagree	2	3	4	5	6	7<br>Strongly<br>Agree

16. In relation to implement physical activity in my nursery/school, when at work with young children, I'm always optimistic about the future.

1<br>Strongly<br>Disagree	2	3	4	5	6	7<br>Strongly<br>Agree

17. If I implement physical activity in my nursery/school, when at work with young children, this will benefit children's health.

1<br>Strongly<br>Disagree	2	3	4	5	6	7<br>Strongly<br>Agree

18. If I implement physical activity in my nursery/school, when at work with young children, it will have disadvantages for my relationship with young children.

1<br>Strongly<br>Disagree	2	3	4	5	6	7<br>Strongly<br>Agree

19. I will definitely implement physical activity in my nursery/school with young children next time I am at work.

1<br>Strongly<br>Disagree	2	3	4	5	6	7<br>Strongly<br>Agree

20. I intend to implement physical activity in my nursery/school with young children next time I am at work.

1<br>Strongly<br>Disagree	2	3	4	5	6	7<br>Strongly<br>Agree

21. How strong is your intention to implement physical activity with young children in your nursery/school next time you are at work?

1<br>Strongly<br>Disagree	2	3	4	5	6	7<br>Strongly<br>Agree

22. I have a clear plan of how I will implement physical activity in my nursery/school when at work with young children.

1<br>Strongly<br>Disagree	2	3	4	5	6	7<br>Strongly<br>Agree

23. I have a clear plan on under what circumstances I will implement physical activity in my nursery/school, when at work with young children.

1<br>Strongly<br>Disagree	2	3	4	5	6	7<br>Strongly<br>Agree

24. I have a clear plan when I will implement physical activity in my nursery/school, when at work with young children.

1<br>Strongly<br>Disagree	2	3	4	5	6	7<br>Strongly<br>Agree

25. I have a clear plan on how often I will implement physical activity in my nursery/school, when at work with young children.

1	2	3	4	5	6	7
Strongly Disagree						Strongly Agree

26. Generally in your nursery/school, when at work with children, how often is covering something else on your agenda a higher priority than implementing physical activity?

1	2	3	4	5	6	7
Never						Always

27. Generally in your nursery/school, when at work with children, how often does covering something else on your agenda take precedence over implementing physical activity?

1	2	3	4	5	6	7
Never						Always

28. Generally in your nursery/school, when at work with children, how often is covering something else on your agenda more urgent than implementing physical activity?

1	2	3	4	5	6	7
Never						Always

29. Generally in your nursery/school, when at work with children, how often is covering something else on your agenda more pressing than implementing physical activity?

1	2	3	4	5	6	7
Never						Always

30. How often do you forget to implement physical activity in your nursery/school, when at work with young children?

1	2	3	4	5	6	7
Never						Always

31. When I need to concentrate on implementing physical activity in my nursery/school, when at work with young children, I have no trouble focusing my attention.

1	2	3	4	5	6	7
Strongly Disagree						Strongly Agree

32. When trying to focus my attention on implementing physical activity in my nursery/school, when at work with young children, I have difficulty blocking out distracting thoughts.

1<br>Never	2	3	4	5	6	7<br>Always

33. When concentrating on implementing physical activity in my nursery/school, when at work with young children, I can focus my attention so that I become unaware of what's going on around me.

1<br>Never	2	3	4	5	6	7<br>Always

34. Within the social-political context there is sufficient financial support (e.g. local authorities, government) for implementing physical activity guidelines in early years settings.

1<br>Strongly<br>Disagree	2	3	4	5	6	7<br>Strongly<br>Agree

35. Within the socio-political context there are good networks between parties involved in guidelines for implementing physical activity in nursery/school settings.

1<br>Strongly<br>Disagree	2	3	4	5	6	7<br>Strongly<br>Agree

36. Most people who are important to me think that I should implement physical activity in my nursery/school, when at work with young children.

1<br>Strongly<br>Disagree	2	3	4	5	6	7<br>Strongly<br>Agree

37. Most people whose opinion I value would approve me to implement physical activity in my nursery/school, when at work with young children.

1<br>Strongly<br>Disagree	2	3	4	5	6	7<br>Strongly<br>Agree

38. Have you recently, during the past two weeks, been able to enjoy your normal day-to-day activities?

1	2	3	4	5	6	7
Never						Always

39. Have you recently, during the past two weeks, been feeling unhappy and depressed?

1	2	3	4	5	6	7
Never						Always

40. I have a clear plan of how I will implement physical activity in my nursery/school, when at work with young children.

1	2	3	4	5	6	7
Strongly Disagree						Strongly Agree

41. I have a clear plan on under what circumstances I will implement physical activity in my nursery/school with young children.

1	2	3	4	5	6	7
Strongly Disagree						Strongly Agree

42. I have a clear plan when I will implement physical activity in my nursery/school with young children.

1	2	3	4	5	6	7
Strongly Disagree						Strongly Agree

43. I have a clear plan on how often I will implement physical activity in my nursery/school, when at work with young children.

1	2	3	4	5	6	7
Strongly Disagree						Strongly Agree

44. I can see how promoting physical activity differs from usual ways of working at my nursery/school.

1<br>Strongly<br>Disagree	2	3	4	5	6	7<br>Strongly<br>Agree

45. I believe that promoting physical activity at my nursery/school is a legitimate part of my role.

1<br>Strongly<br>Disagree	2	3	4	5	6	7<br>Strongly<br>Agree

46. Sufficient resources are available at my nursery/school to support promoting physical activity.

1<br>Strongly<br>Disagree	2	3	4	5	6	7<br>Strongly<br>Agree

47. Management at my nursery/school adequately support promoting physical activity.

1<br>Strongly<br>Disagree	2	3	4	5	6	7<br>Strongly<br>Agree

48. Promoting physical activity fits well with strategic goals of my nursery/school.

1<br>Strongly<br>Disagree	2	3	4	5	6	7<br>Strongly<br>Agree

49. Staff in my nursery/school have a shared understanding of the purpose of promoting physical activity.

1<br>Strongly<br>Disagree	2	3	4	5	6	7<br>Strongly<br>Agree

50. Staff in my nursery/school agree that promoting physical activity in early years is worthwhile.

1<br>Strongly<br>Disagree	2	3	4	5	6	7<br>Strongly<br>Agree

Thank you for your collaboration!
