# Supplementary material for: Early Years Physical Activity and Motor Skills Intervention—A Feasibility Study to Evaluate an Existing Training Programme for Early Years Educators
Source: Children (Basel). 2023 Jan 11;10(1):145. doi: 10.3390/children10010145 (PMC9856565; doi:10.3390/children10010145)
Supplement: Supplementary file 1 [file children-10-00145-s001.zip › children-2097562-Supplementary tables S1-S3.pdf]

Supplementary Table S1. Observations of Educator behaviour at baseline

Baseline observation	Intervention		Control	
**Activity**	**Observed**	**n**	**Observed**	**n**
Educators plan the movement activities beforehand	No<br>Yes	2<br>3	No<br>Yes	3<br>0
Educators promote activities that encourage children to move around	No<br>Yes	1<br>4	No<br>Yes	0<br>3
Children are encouraged to play outside	No<br>Yes	1<br>4	No<br>Yes	1<br>2
Educators promote activities that encourage children to run	No<br>Yes	2<br>3	No<br>Yes	3<br>0
Educators promote activities that encourage children to dance	No<br>Yes	1<br>4	No<br>Yes	1<br>2
Educators promote activities that encourage children to hop and jump in different ways	No<br>Yes	2<br>3	No<br>Yes	3<br>0
Children are taught about the different ways to jump	No<br>Yes	2<br>3	No<br>Yes	3<br>0
Educators promote activities that give children the opportunity to be involved in challenging movement situations which involves coordination and balance	No<br>Yes	2<br>3	No<br>Yes	2<br>1
Educators guide children on activities that involve coordination and balance	No<br>Yes	2<br>3	No<br>Yes	2<br>1

Educators promote activities that encourage children to throw and catch	No<br>Yes	1<br>4	No<br>Yes	2<br>1
Children are taught how to throw and catch	No<br>Yes	1<br>4	No<br>Yes	2<br>1
Educators promote activities that give children the opportunity to skip	No<br>Yes	4<br>1	No<br>Yes	3<br>0
Children are taught how to skip	No<br>Yes	3<br>2	No<br>Yes	3<br>0
Educators try to engage all children in the activity	No<br>Yes	1<br>4	No<br>Yes	1<br>2

Supplementary Table S2. Results of the questionnaire measuring Educator behavioural predictors at baseline (completed by Intervention Educators only, n=10).

Domain*	Median	Minimum	Maximum
Knowledge	5.3	2.5	6.8
Skills	4.5	2.3	7.0
Social/Professional role & identity	6.6	5.8	7.0
Belief about Capabilities	5.3	4.3	7.0
Optimism	6.0	4.0	7.0
Beliefs about consequences	7.0	3.5	]7.0
Intentions	7.0	5.3	7.0
Goals - Action Planning	5.3	2.0	7.0
Goals - priority	4.8	2.0	7.0
Memory, attention & decision process	5.1	3.8	6.0
Environmental context and resources	3.8	1.0	6.0
Social influences	6.0	4.0	7.0
Emotion	5.3	4.5	7.0
Behavioural regulation	5.5	2.5	7.0
Social and professional role	4.8	4.0	6.0
Work Environment	6.1	2.4	7.0

*Answers ranged on a scale of 1-7, where 1=strongly disagree/never, and 7=strongly agree/always; mean taken for all questions in the domain.

Supplementary Table S3. Participants' characteristics (children) at baseline (N=43)

	**Baseline**		**Follow-up**	
	**Intervention (n=30) Mean ± SD**	**Control (n=13) Mean ± SD**	**Intervention (n=23) Mean ± SD**	**Control (n=5) Mean ± SD**
Age (years)	3.6 ± 0.7	3.5 ± 0.5	-	-
Gender (% female)	40	61.5	-	-
Anthropometrics				
Height (cm)	101.6 ± 6.6	99.0 ± 6.1	107.3 ± 6.1	99.3 ± 2.5
Weight (kg)	16.7 ± 2.4	16.7 ± 2.9	18.6 ± 3.2	16.0 ± 1.8
BMI z-score	0.20 ± 1.17	0.1 ± 1.4	0.61 ± 0.91	0.37 ± 0.86
Fundamental Motor Skills				
FMS total score	72.7 ± 15.7	59.9 ± 24.1	77.0 ± 13.0	72.4 ± 8.1
FMS standard score	9.0 ± 3.4	6.9 ± 4.5	9.9 ± 3.1	8.8 ± 1.9
FMS percentile	41.5 ± 33.6	28.4 ± 34.3	47.4 ± 31.2	35.6 ± 23.3
Physical Activity (ActivPAL)	**n=21**	**n=6**	**n=15**	**n=0**
Time sitting (%)	43.8 ± 10.6	36.3 ± 14.1	55.5 ± 10.8	-

Time standing (%)	33.4 ± 8.6	38.3 ± 10.9	30.0 ± 8.2	-
Time stepping (%)	22.8 ± 6.0	25.4 ± 6.3	14.5 ± 4.6	-

Supplementary Table S4. Intervention group comparison follow-up – baseline

	**Mean difference (95% CI)**	**P value**
Child anthropometrics (n=23)		
Weight (kg)	1.6 (1.0 to 2.2)	<0.0001
BMI z-score	0.14 (-0.31 to 0.59)	0.516
Fundamental Motor Skills (n=23)		
FMS total score	3.8 (-2.8 to 10.4)	0.249
FMS standard score	0.8 (-0.7 to 2.3)	0.281
FMS percentile	4.8 (-9.0 to 18.6)	0.481
Physical Activity (ActivPAL) (n=7)		
Time sitting (%)	10.8 (-3.9 to 25.5)	0.122
Time standing (%)	-2.8 (-11.6 to 6.0)	0.466
Time stepping (%)	-8.0 (-14.4 to -1.6)	0.022
